# Sociodemographic determinants, clinical characteristics, and survival outcomes of solid tumor patients at Tikur Anbessa Specialized Hospital, Ethiopia: a five-year retrospective cohort study

**DOI:** 10.21203/rs.3.rs-7797194/v1

**Published:** 2025-11-20

**Authors:** Mahlet Moges, Legese Chelkeba, Ephrem Abebe, Abdella Birhan Yabeyu, Shivan A. Patel, Mohammed K. Ali

**Affiliations:** 1 Department of Pharmacology and Clinical Pharmacy, School of Pharmacy, College of Health Sciences, Addis Ababa University, Addis Ababa, Ethiopia.; 2 Department of Pharmacy Practice, Purdue University, USA; 3 Department of Pharmacy, College of Medicine and Health Sciences, Ambo University, Ambo, Ethiopia; 4 Hubert Department of Global Health, Rollins School of Public Health, Emory University, Georgia, Atlanta, USA.

**Keywords:** solid tumors, survival, prognostic factors, Ethiopia, Tikur Anbessa Specialized Hospital

## Abstract

**Background:**

Cancer survival in sub-Saharan Africa is poorly characterized, and Ethiopia lacks large-scale outcome data. Tikur Anbessa Specialized Hospital (TASH), the country’s only comprehensive cancer center, receives the majority of referrals, yet survival outcomes and determinants remain underreported. This study evaluated sociodemographic determinants, clinical characteristics, and survival outcomes of patients with solid tumors treated at TASH.

**Methods:**

We conducted a five-year retrospective cohort study including 1,127 adults diagnosed with histologically confirmed solid tumors at TASH between September 2016 and December 2017. Patients were followed until death or censoring at 60 months. Sociodemographic and clinical data were abstracted from medical records. Survival was estimated using Kaplan–Meier methods and compared by log-rank tests. Multivariable Cox regression identified independent predictors of mortality.

**Results:**

The median age at diagnosis was 47 years; 65.9% were female, and 47.4% had no formal education. Cervical (21.5%), breast (17.3%), and colorectal (9.1%) cancers were most frequent. Almost half of patients (49.2%) presented with stage IV disease. Over five years, 832 deaths occurred, yielding a case fatality of 73.8%. The median overall survival was 10 months (95% CI: 8.96–11.04), and the 5-year survival rate was 26.2%. Early-stage disease conferred significantly longer survival (26 vs. 8 months; p < 0.001). Patients receiving multimodal therapy had a median survival of 21 months, compared with 3 months among those without chemotherapy. Hormonal therapy (median 24 months) and paclitaxel-based regimens (20 months) were associated with the longest survival. Independent predictors of higher mortality included advanced stage (AHR 2.02, 95% CI: 1.53–2.68), being single (AHR 1.41, 95% CI: 1.11–1.80), carcinogen exposure (AHR 2.35, 95% CI: 1.57–3.53), complications (AHR 1.23, 95% CI: 1.05–1.45), and receipt of palliative care (AHR 1.82, 95% CI: 1.38–2.39).

**Conclusion:**

Solid tumor patients at TASH experience alarmingly poor outcomes, primarily due to late-stage presentation and limited treatment access. Strengthening early detection, expanding multimodal therapy, and addressing social determinants are critical for improving survival in Ethiopia.

## 1. Background

The Global Burden of Disease 2019 study estimated 23.6 million new cancer cases and 10 million deaths worldwide, representing a more than 20% increase since 2010(1). Although high-income countries accounted for the largest absolute number of cases, the steepest percentage increases occurred in low- and middle-income countries (LMICs) (1). Advances in prevention, early detection, and treatment have improved outcomes in high-income settings, but LMICs remain disproportionately affected (2–4). In sub-Saharan Africa, and particularly in Ethiopia, cancer control efforts are constrained by fragile health systems, limited diagnostic and treatment infrastructure, and pronounced sociodemographic disparities. These challenges contribute to delayed presentation, advanced-stage diagnosis, and poor survival (5). Population-based registry data from Ethiopia confirm that most cancers are detected late, reflecting restricted access to pathology and imaging services as well as limited community awareness (6). Shortages of trained oncology professionals, radiotherapy machines, and essential medicines further compromise the quality and timeliness of care (7). Over 40% of Ethiopia’s population is under 15 years (6), highlighting a demographic at risk for both pediatric and adult malignancies, yet comprehensive survival data remain scarce. Sociodemographic factors such as socioeconomic status, education, and geographic residence are increasingly recognized as major drivers of cancer outcomes. Evidence from LMICs underscores these disparities: in Ethiopia, lower socioeconomic and educational status has been linked with advanced-stage presentation among breast and cervical cancer patients (8), while studies from the East African corridor confirm the impact of socioeconomic deprivation on esophageal cancer risk (9).

The cancer profile in Ethiopia differs from that of high-income countries. Cervical cancers (28.9%), breast cancer (22.7%), nasopharyngeal carcinoma (7.6%), and colorectal cancer (7.1%) predominate [7,9], whereas lung and prostate cancers are more frequent globally(10). These variations likely reflect unique genetic, infectious, and environmental factors, compounded by diagnostic limitations (11–14). Despite the known impact of social determinants and clinical features, no comprehensive Ethiopian cohort study has systematically examined their role in survival across solid tumors. Current evidence is limited to single-disease reports or descriptive hospital-based audits (6,15,16).

Critical knowledge gaps include the influence of sociodemographic status on survival, patterns of stage at presentation, treatment adherence, and unmet supportive care needs (17). Without this evidence, the design of equitable, evidence-based cancer control strategies remains severely constrained. By comprehensively examining determinants of survival across major solid tumors, this study will fill a critical evidence gap. The findings will inform national cancer control planning, improve patient outcomes, and contribute to reducing global cancer inequities. Ultimately, addressing these disparities is essential for advancing equity in cancer care, both in Ethiopia and across comparable LMIC settings.

## 2. Methods

### 2.1. Study Area

This study was conducted at TASH in Addis Ababa, Ethiopia, the country’s largest tertiary referral and teaching hospital. Established in the early 1960s, TASH serves as the national oncology hub, providing chemotherapy, radiotherapy, surgery, pain management, supportive care, and palliative services. It also functions as a central site for cancer registration, early detection initiatives, and the development of standardized treatment protocols.

### 2.2. Study Design and Period

We employed a retrospective cohort design based on medical record review. The cohort included patients diagnosed with solid tumors at TASH between January 2016 and February 2017. Data abstraction was carried out between July 2021 and February 2022.

### 2.3. Study Population

The source population comprised all cancer patients treated at TASH during the study period. Eligible patients were:

- Aged ≥14 years (adolescent and adult services are integrated in Ethiopia).
- Diagnosed with histologically confirmed solid tumors.
- Possessing complete medical records, and
- Available for survival verification via records or telephone follow-up.

Exclusion criteria included: incomplete records, refusal of follow-up by telephone for ascertaining patient outcome (death vs. alive), and hematologic malignancies, due to their distinct biology, treatment, and survival trajectories.

### 2.4. Sample Size and Sampling Procedure

All eligible medical records during the study period were included. Of 1,546 identified cases, 419 were excluded due to missing or incomplete data, yielding 1,127 patients for final analysis.

### 2.5. Study Variables

The selection of study variables was guided by both theoretical and empirical considerations. The Andersen Behavioral Model of Health Service Use and the Socioecological Model provided the conceptual basis, highlighting how demographic, socioeconomic, geographic, clinical, treatment-related, and behavioral factors jointly influence health outcomes, treatment patterns, and survival in cancer patients. Demographic and socioeconomic variables (age, sex, marital status, education, occupation) were included because they are well-documented determinants of cancer incidence, stage at diagnosis, and survival. Prior studies in oncology and health disparities research consistently show these factors affect access to care, treatment adherence, and prognosis. Clinical variables (cancer type, stage, comorbidities, complications) were selected based on their direct relevance to disease progression and survival probabilities, supported by multiple epidemiologic studies. Treatment variables (chemotherapy use, other medications, herbal medicine) reflect both biomedical and traditional care pathways, allowing exploration of treatment patterns relevant to LMIC settings. Behavioral variables (alcohol, tobacco, khat use) were included as modifiable risk factors known to affect treatment outcomes and cancer progression. Variable inclusion was also determined by data availability within the cancer registry and patient records. Some potentially relevant factors (e.g., nutritional status, genetic markers, or healthcare facility type) were unavailable or incomplete and thus not included in the final analysis.

### 2.6. Data Collection Procedures

Data were collected using a structured English-language checklist adapted from prior studies and pretested on 50 patients (5% of the sample). Trained oncology health professionals performed data abstraction under supervision. Information captured included demographic and socioeconomic characteristics, tumor type and stage, comorbidities, treatment modalities, chemotherapy agents, co-medications, substance use, complications, and survival status. Survival data were obtained from medical records when available, or via telephone follow-up.

### 2.7. Data Management and Quality Assurance

The checklist was pretested for clarity, and data collectors were trained. Daily supervision, cross-checking, and validation were performed to ensure completeness and consistency. Data were coded, cleaned, and entered into SPSS version 26 for analysis.

### 2.8. Data Analysis

Descriptive statistics (frequencies, proportions, means, medians) summarized patient, disease, and treatment characteristics. Kaplan–Meier methods with life tables estimated median and overall survival, and log-rank tests compared survival distributions across variables. Multivariable Cox proportional hazards regression was used to identify independent predictors of mortality. Variables with *p* < 0.20 in bivariable analysis were included in multivariable models. The proportional hazards assumption was assessed using Schoenfeld residuals; *p* > 0.05 indicated validity. Statistical significance was set at *p* < 0.05.

### 2.9. Operational Definitions

- Event: death from any cause during follow-up.
- Censored: alive, lost to follow-up, or alive at study end.
- Comorbidity: any documented non-cancer medical condition.
- Substance use: reported use of tobacco, khat, or alcohol.
- Palliative care: symptom management provided for incurable disease, as documented in records.
- Time to event: months from date of diagnosis to death or censoring.
- Stage at diagnosis: based on Tumor–Node–Metastasis (TNM) classification of solid tumors.

## 2. Results

### 2.1. Study Population and Follow-up

**Figure 1:**
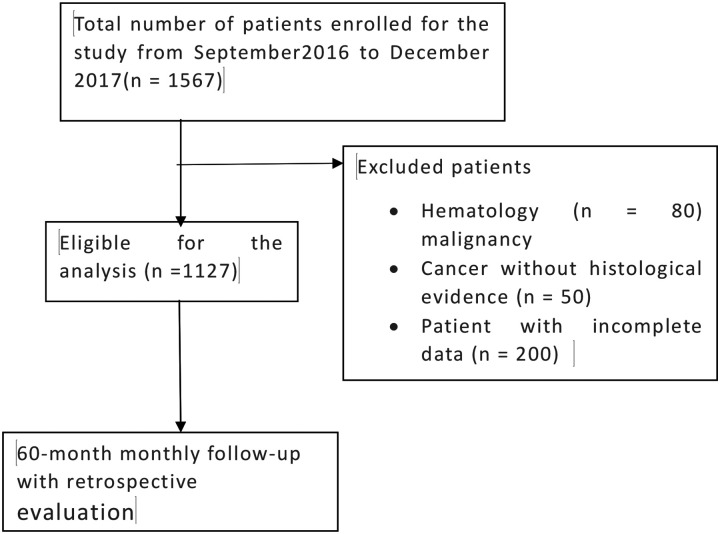
Figure of flow chart for data extraction criteria in TASH

Between September 2016 and December 2017, a total of 1,567 patients were enrolled in the study. Of these, 1,127 patients met eligibility criteria and were included in the analysis. Exclusions comprised hematologic malignancies (n = 80), cancers without histological confirmation (n = 50), and incomplete records (n = 200. All included patients were followed monthly for up to 60 months using retrospective data collection (Figure 1).

### 2.2. Socio-Demographic Characteristics of Study Participants

**Figure 2:**
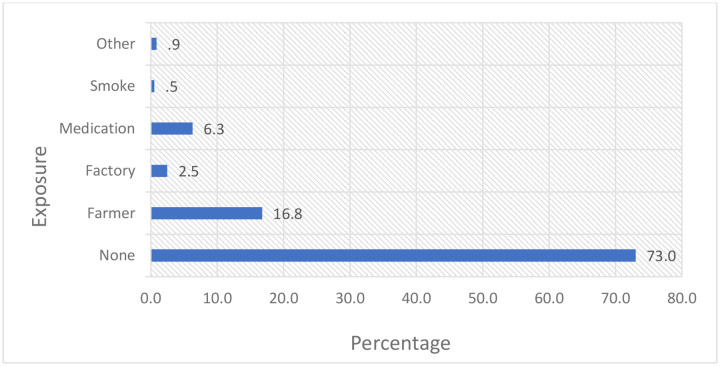
Frequency of patient status with type of occupational exposure for cancer patient in TASH (N = 1127). Other: Asbestos (3), Obesity (3) and coal tar (4).

**Figure 3:**
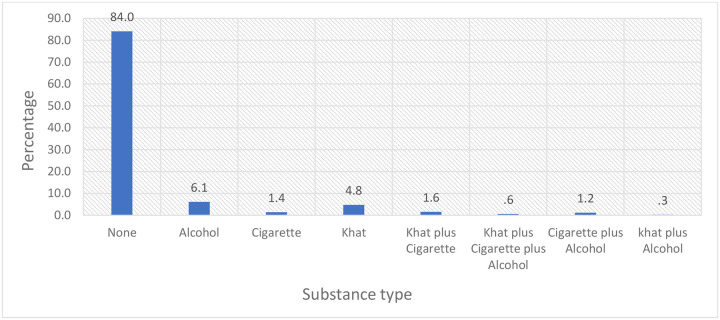
Frequency of patient status with the type of substance use in TASH (N = 1127)

The median age of the included patients was 47 years, with the majority (45.2%) falling within the 40–59-year age range. Females predominated (65.9%). Patients mainly originated from Oromia (32.1%), Addis Ababa (28.3%), and Amhara (19.5%). The majority were married (62.6%), and educational attainment was low, with nearly half (47.4%) being illiterate. Unemployment was common (42.3%), and 27% reported occupational exposure to carcinogens. Farming was the most common identified exposure (16.8%), followed by medications (6.3%) and factory work (2.5%). Treatment costs were largely subsidized by the government (58.4%), while 41.6% of patients paid out-of-pocket. Substance use was reported by 16% of patients, primarily alcohol consumption (6.1%) and khat chewing (4.8%) (**Table**, Figure 2 and 3)

### 2.3. Distribution of Cancer Types Among the Study Population

**Figure 4:**
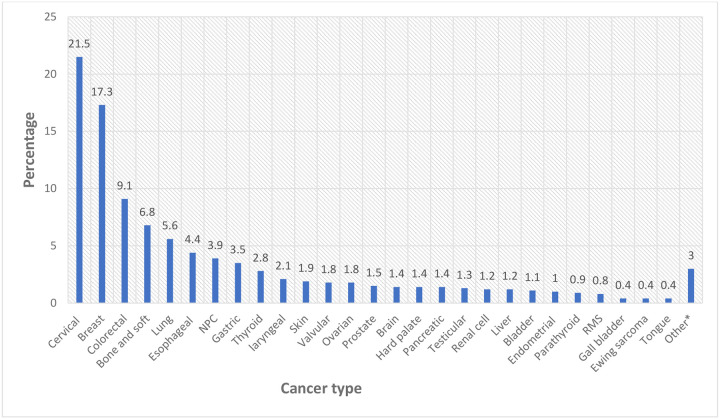
Spectrum of cancer and their frequency at TASH (N = 1127) **Abbreviations:** NPC – Nasopharyngeal carcinoma; RMS – Rhabdomyosarcoma. Other*: Carotid body tumor (3), sinonasal tumor (4), maxillary tumor (4), hypopharyngeal tumor (4), gingival tumor (1), small bowel tumor (2), gastrojejunal tumor (1), penile cancer (3), sublingual cancer (1), tracheal tumor (1), thymic tumor (2), conjunctival tumor (1), Kaposi sarcoma (3).

Cervical cancer was the most frequently diagnosed malignancy, accounting for 242 cases (21.5%), followed by breast cancer with 195 cases (17.3%) and colorectal cancer with 103 cases (9.1%). Other common malignancies included bone and soft tissue tumors (77, 6.8%), lung cancer (63, 5.6%), and esophageal cancer (50, 4.4%). Nasopharyngeal carcinoma, gastric cancer, and thyroid cancer represented 3.9%, 3.5%, and 2.8%, respectively. Less frequent cancers included laryngeal (24, 2.1%), skin (21, 1.9%), valvular (20, 1.8%), ovarian (20, 1.8%), and prostate cancer (17, 1.5%). Rare malignancies, each accounting for less than 1.5% of cases, included brain, hard palate, pancreatic, testicular, renal cell, liver, bladder, endometrial, parathyroid cancers, rhabdomyosarcoma (RMS), gall bladder, Ewing sarcoma, and tongue cancers (Figure 4).

### 2.4. Distribution of Cancer Types by Sex

**Figure 5:**
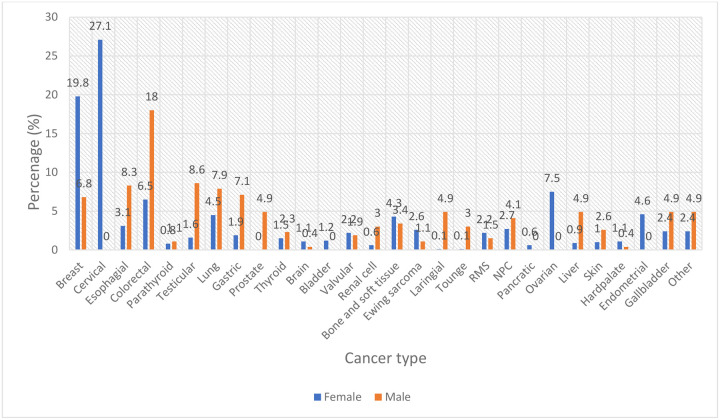
Frequency of type of cancer by gender categories in TASH oncology center of solid tumor. NPC – Nasopharyngeal carcinoma; RMS – Rhabdomyosarcoma

Among the female patients (n = 743), cervical cancer was the most prevalent malignancy, accounting for 27.1% of cases, followed by breast cancer (19.8%) and ovarian cancer (7.5%). Other notable cancers included endometrial (4.6%), bone and soft tissue tumors (4.3%), and colorectal cancer (6.5%). Less common malignancies among females were esophageal (3.1%), valvular (2.2%), and rhabdomyosarcoma (2.2%), with all remaining cancer types comprising less than 2% each. In contrast, among male patients (n = 384), colorectal cancer was the most frequently diagnosed malignancy (18.0%), followed by testicular cancer (8.6%) and lung cancer (7.9%). Gastric (7.1%), liver (4.9%), prostate (4.9%), gallbladder (4.9%), and nasopharyngeal carcinoma (4.1%) were also observed with moderate frequency. Other malignancies, including esophageal (8.3%), thyroid (2.3%), and rare tumors such as tongue (3.0%) and laryngeal cancer (4.9%), were less common. Overall, the distribution of cancers demonstrates a clear sex-specific pattern, with gynecological malignancies predominating in females, whereas gastrointestinal, genitourinary, and head-and-neck tumors were more frequent in males (Figure 5).

### 2.5. Clinical Characteristics of the Study Population

Nearly half of patients (49.2%) presented with stage IV cancer, underscoring late diagnosis as a critical challenge. Comorbidities were reported in one-fifth (19.4%), most commonly HIV (5.8%) and hypertension (4.2%). About 30% of patients were on medications, with HAART (5.9%) and herbal therapies (10.0%) predominating. Complications affected 24.5% of patients, with pleural effusion, paralysis, thrombosis, and renal problems being most frequent. Tumor recurrence occurred in 12.1% during follow-up (Table 2).

### 2.6. Treatment Modalities

**Figure 6.**
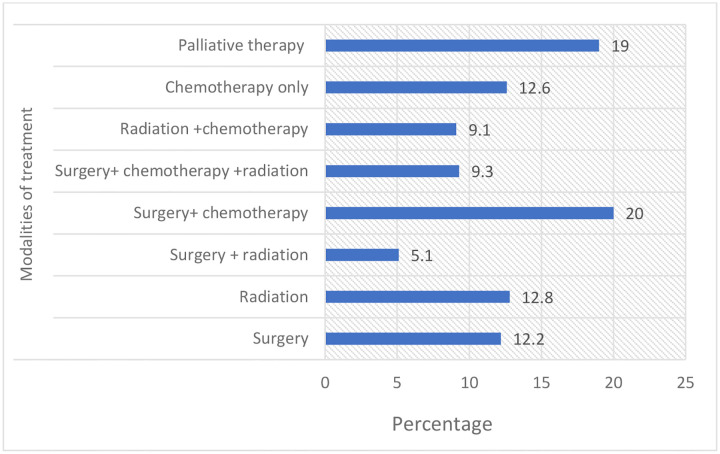
Distribution of treatment modalities among cancer patients TASH.

Among the cohort, treatment strategies showed considerable variation. The most common was combination therapy with surgery and chemotherapy (20.0%, n = 225), followed by palliative care (19.0%, n = 214). Single-modality approaches included chemotherapy (12.6%, n = 142), radiation (12.8%, n = 144), and surgery (12.2%, n = 137). Multimodality regimens were also employed: radiation plus chemotherapy (9.1%, n = 102), surgery plus radiation (5.1%, n = 58), and trimodality therapy combining surgery, chemotherapy, and radiation (9.3%, n = 105) (Figure 6).

### 2.7. Chemotherapy Regimens

**Figure 7:**
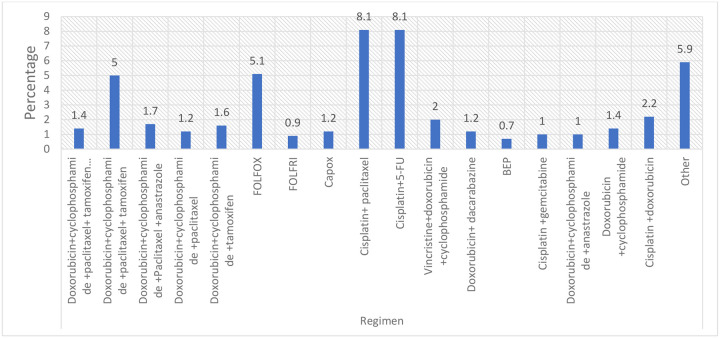
Regimen used in the treatment of cancer patients at TASH. **Abbreviation**: 5-Fluorouracil (5-FU); 5-Fluorouracil, leucovorin, and oxaliplatin (FOLFOX); 5-Fluorouracil, leucovorin, and irinotecan (FOLFIRI); capecitabine and oxaliplatin (CAPOX); bleomycin, etoposide, and cisplatin (BEP); 5-Fluorouracil, doxorubicin, and cyclophosphamide (FAC); and ifosfamide plus etoposide (IE). **Other**: cisplatin alone (n = 15), tamoxifen alone (n = 10), anastrozole alone (n = 9), FAC (n = 6), cisplatin + paclitaxel + doxorubicin (n = 3), cisplatin + etoposide + paclitaxel (n = 4), unknown chemotherapy (n = 10), etoposide + cisplatin (n = 5), docetaxel alone (n = 2), IE (n = 3), cyclophosphamide + epirubicin (n = 2), and vinorelbine (n = 2).

A wide range of chemotherapy regimens was utilized among the study population. The most frequently administered treatments were platinum- and taxane-based combinations, notably cisplatin plus paclitaxel (8.1%) and cisplatin plus 5-fluorouracil (8.1%), followed by FOLFOX (5.1%) and other combination regimens (5.9%). Anthracycline-based combinations, including doxorubicin plus cyclophosphamide with or without paclitaxel and adjuvant hormonal therapy, were used less frequently, ranging from 1.4% to 5.0%. Additional regimens, each administered to fewer than 2% of patients, included FOLFIRI, CAPOX, vincristine plus doxorubicin plus cyclophosphamide, doxorubicin plus dacarbazine, BEP, cisplatin plus gemcitabine, and cisplatin plus doxorubicin (Figure 7).

### 2.8. Pattern of Chemotherapy Use

**Figure 8:**
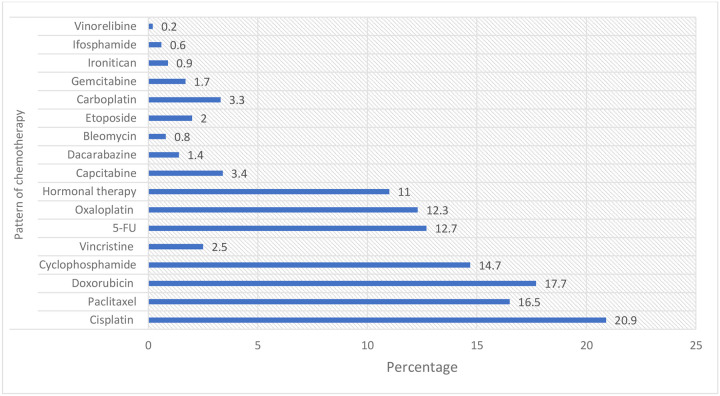
Pattern of chemotherapy use among cancer patients at TASH. 5-FU: 5-Florouracil

Among the chemotherapy regimens administered, doxorubicin (17.7%), paclitaxel (16.5%), cisplatin (20.9%), and cyclophosphamide (14.7%) were the most frequently utilized agents. Other commonly used drugs included 5-fluorouracil (12.7%), oxaliplatin (12.3%), and hormonal therapy (11%). Less frequently administered agents comprised carboplatin (3.3%), capecitabine (3.4%), etoposide (2%), vincristine (2.5%), gemcitabine (1.7%), dacarbazine (1.4%), irinotecan (0.9%), ifosfamide (0.6%), bleomycin (0.8%), and vinorelbine (0.2%) (Figure 8).

### 2.9. Overall, 5-year survival rates of patients

In this cohort of 1,127 patients with diverse solid malignancies followed for five years, 832 deaths occurred, corresponding to an overall case-fatality proportion of 73.8%. Among the 295 survivors, 240 (21.3%) were classified as cured based on histopathology and follow-up telephone call, while 55 (4.9%) continued palliative care despite advanced disease (Figure 9).

**Figure-10:**
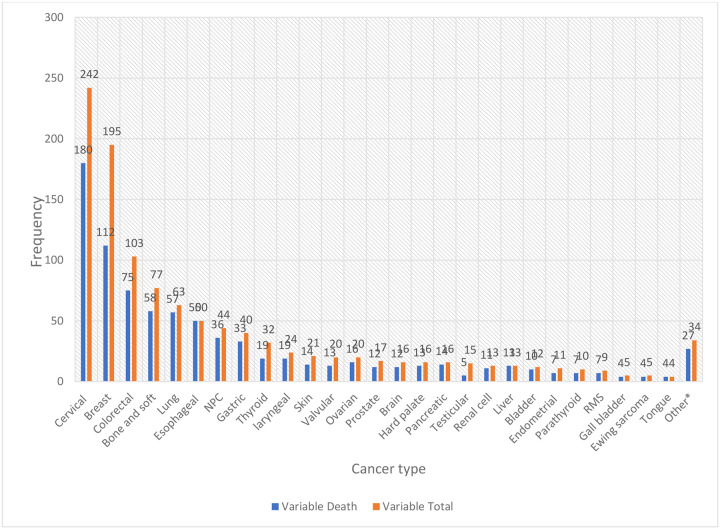
Frequency of mortality by cancer type at TASH. NPC- Nasopharyngeal Cancer, RMS-Rhabdomyosarcoma. Other*: Carotidbodytumor (3), Sinonasal (4) Maxillary (4) Hypopharyngeal (4) Gingival (1) Smallball tumor (2), Gastrojuoginaltumor (1), penial cancer (3), Sublingual cancer (1), Tracheal (1), Thymus (2), Conjunctivital (1), Kaposi sarcoma (3)

Mortality was driven largely by common and aggressive cancers: cervical cancer accounted for the largest share of deaths (180/835; 21.6%), followed by breast cancer (112/835; 13.5%), reflecting both incidence and relative survival. Several less frequent malignancies demonstrated near-universal lethality, including oesophageal (50/50; 100%) and hepatic (13/13; 100%) cancers. High case-fatality proportions were also observed in pancreatic (87.5%), lung (90.5%), gastric (82.5%), nasopharyngeal (81.8%), ovarian (80.0%), renal cell (84.6%), and bladder (83.3%) cancers. Conversely, indolent malignancies such as thyroid (59.4%) and testicular (33.3%) cancers showed markedly lower fatality, consistent with more favorable biology and therapeutic responsiveness (Figure 10).

### 2.10. Survival Analysis

The overall median survival time was 10 months (95% CI: 8.96–11.04). Survival did not differ significantly by marital status (log-rank p = 0.054), although divorced patients had the longest median survival (12 months) and single patients the shortest (8 months). Exposure to the assessed risk factor showed no significant association (11 vs. 8 months; p = 0.181). Survival was strongly influenced by cancer stage: early-stage disease (Stage I–II) was associated with a median survival of 26 months versus 8 months in advanced-stage disease (Stage III–IV; p < 0.001). Patients with recurrence had longer median survival (18 vs. 9 months; p < 0.001). Complications were associated with a modest reduction (9 vs. 10 months; p = 0.0126).

Treatment modality significantly affected outcomes (p < 0.001). Multimodal therapy (surgery + chemotherapy + radiotherapy) yielded the longest median survival (21 months), whereas absence of chemotherapy was associated with the shortest (3 months). Chemotherapy agent type also mattered: paclitaxel uses improved survival (20 vs. 8 months; p < 0.001), as did doxorubicin (16 vs. 9 months; p < 0.001). Hormonal therapy conferred the greatest survival benefit, with treated patients achieving a median survival of 34 months compared to 9 months among non-users (p < 0.001) (Table 3).

**Figure 11:**
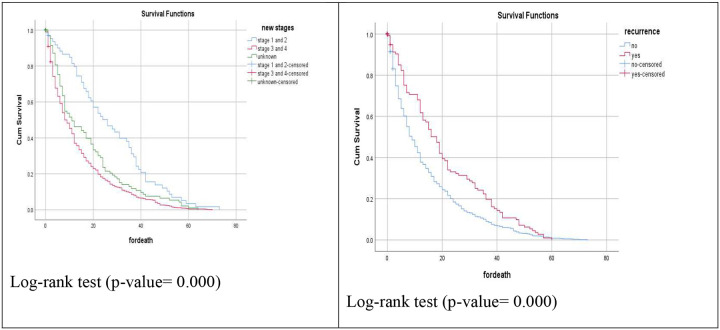

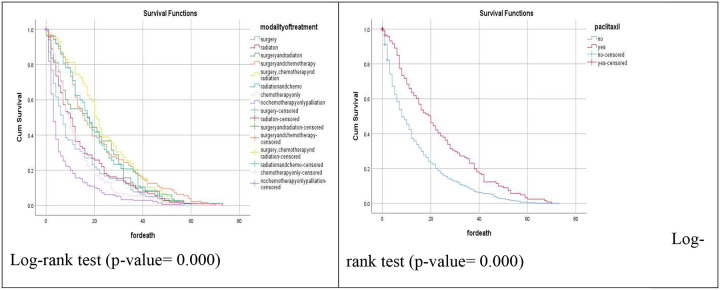
Kaplan-Meier survival analysis curve in variables showed significant association in terms of survival of TASH

A Kaplan–Meier survival analysis revealed statistically significant differences in patient survival across key clinical variables, including cancer stage, treatment modality, and the use of specific therapies such as paclitaxel and hormonal agents (Figure 11).

### 2.11. Multivariable Predictors of Mortality

In this five-year cohort of 1,127 adults with solid tumors, multivariable Cox regression identified several independent predictors of mortality. Patients with advanced-stage disease (Stage III–IV) had a twofold higher risk of death compared to those with early-stage tumors (AHR 2.02, 95% CI 1.53–2.68, p < 0.001). Other adverse prognostic factors included single marital status (AHR 1.41, 95% CI 1.11–1.80, p = 0.006), history of relevant exposures (AHR 2.35, 95% CI 1.57–3.53, p < 0.001), presence of complications (AHR 1.23, 95% CI 1.05–1.45, p = 0.012), and receipt of palliative treatment (AHR 1.82, 95% CI 1.38–2.39, p < 0.001).

Conversely, survival was significantly improved by multimodal therapies. Patients treated with surgery plus chemotherapy (AHR 0.67, 95% CI 0.48–0.95, p = 0.026) and radiation plus chemotherapy (AHR 0.68, 95% CI 0.46–1.00, p = 0.047) had lower mortality risks compared with those receiving single-modality care. Among individual agents, Paclitaxel (AHR 0.74, 95% CI 0.59–0.94, p = 0.014) and hormonal therapy (AHR 0.59, 95% CI 0.40–0.87, p = 0.008) were associated with reduced mortality, whereas Doxorubicin was linked to an increased hazard of death (AHR 1.57, 95% CI 1.03–2.38, p = 0.035). No significant associations were observed for gender, substance use, hospital costs, or other chemotherapeutic agents (Table 4).

## 3. Discussion

This study provides a comprehensive assessment of cancer epidemiology, treatment patterns, and survival outcomes in a large cohort of Ethiopian patients treated at the country’s largest tertiary referral center. The baseline demographic profile highlights key social determinants of health affecting cancer care in Ethiopia. The cohort was predominantly female (65.9%) and relatively young, with over half aged <50 years, reflecting the high burden of cervical and breast cancers (6,18,19). Socioeconomic vulnerability was substantial: nearly half of patients (47.4%) were illiterate, 42.3% were unemployed or engaged in informal labor, and 41.6% were required to self-finance care. These factors are known to create barriers to early diagnosis, adherence to therapy, and completion of treatment, while increasing the risk of catastrophic health expenditure (20–22). Most patients originated from Oromia, Addis Ababa, and Amhara, regions proximate to the capital, indicating potential underrepresentation of rural populations, who face additional geographic, financial, and cultural barriers to care (23). This is highly consistent with prior Ethiopian and regional research, confirming the younger age at presentation, female predominance, low socioeconomic status, and concentration of patients from urban/proximal regions (24). Importantly, this study adds a large, five-year cohort with quantified survival outcomes, which most previous Ethiopian studies have lacked.

Cervical cancer was the most prevalent malignancy, followed by breast and colorectal cancers. Among women, gynecological malignancies predominated, whereas men were more commonly affected by colorectal, testicular, and lung cancers. This sex-specific distribution mirrors global patterns observed in LMICs, where cervical cancer remains a leading cause of cancer-related mortality among women, often due to inadequate screening and HPV vaccination coverage (25). The prominence of cervical cancer underscores gaps in HPV vaccination, screening, and early detection programs (26). Breast cancer prevalence may reflect increasing incidence associated with urbanization and improved health-seeking behavior among women in urban centers (27). The high incidence of colorectal and other gastrointestinal malignancies among men may reflect dietary, occupational, and environmental exposures that warrant further investigation(28–30). Rare tumors, including brain, pancreatic, and sarcomas, while individually uncommon, collectively contributed meaningfully to the cancer spectrum, highlighting the diversity of oncologic presentations in this population(31).

This cohort reveals substantial gaps in Ethiopia’s oncology continuum. A striking 68.1% of patients presented with advanced disease (Stage III–IV), nearly half of whom had Stage IV disease. Advanced stage at presentation remains the dominant determinant of poor survival and reflects delays in diagnosis (8,32,33). Coexisting conditions, including HIV (5.8%) and tuberculosis (2.5%), further complicate management, illustrating the intersecting burdens of infectious and non-communicable diseases (32,34). Notably, 10% of patients reported using herbal medications, indicating reliance on pluralistic care pathways (35,36). While culturally rooted, such practices may delay presentation, increase the risk of herb–drug interactions, and reduce adherence to evidence-based therapy. Additionally, 24.5% of patients experienced serious treatment-related complications, and 19% received exclusively palliative care, reflecting limited radiotherapy capacity and high financial barriers (33,37). These findings underscore the need for early detection, expanded therapeutic infrastructure, and culturally sensitive educational interventions to improve adherence to evidence-based oncology care.

Only a minority of Ethiopian cancer patients receive multimodality therapy, reflecting systemic constraints in oncology service delivery. For instance, in a large cervical cancer cohort at TASH, just 22.5% of patients received concurrent chemoradiation, while the remainder were treated with radiotherapy alone due to prolonged wait times, drug unavailability, or comorbidities. Radiotherapy waiting times often exceeded five months, leading to disease progression in some patients(34,38). These findings underscore the bottlenecks created by limited radiotherapy capacity, long waiting lists, and financial and logistical barriers.

Surgery and chemotherapy remain the mainstay of curative-intent therapy in Ethiopia, but their delivery is frequently constrained by insufficient pathology services, absence of immunohistochemistry and molecular profiling, recurrent drug stockouts, and inadequate supportive care (37,39,40). Cisplatin-, doxorubicin-, and paclitaxel-based regimens are the most commonly administered and are generally aligned with global standards for cervical and breast cancer; however, carboplatin, oral agents, targeted therapies, and immunotherapies remain largely unavailable (41). As a result, dose reductions, treatment delays, and empiric regimens are frequent, reducing therapeutic intensity and potentially compromising outcomes (37). Collectively, these findings highlight an implementation gap rather than a knowledge gap among Ethiopian oncologists, pointing to the urgent need for strategic formulary expansion, investment in diagnostic capacity, integration of supportive care, and radiotherapy infrastructure scale-up (7).

Mortality patterns reflected both the high incidence of cervical and breast cancers and the aggressive biology of certain rare tumors. Cervical cancer accounted for the largest proportion of deaths, consistent with LMIC trends driven by delayed diagnosis, limited screening, and suboptimal HPV vaccination coverage (42). Breast cancer contributed less to mortality relative to incidence, consistent with evidence that systemic therapy and surgery substantially improve survival even in constrained radiotherapy contexts (25). Tumors with inherently poor prognosis, including esophageal, hepatic, pancreatic, gastric, and lung cancers, demonstrated very high case-fatality rates, approaching 100% for esophageal and hepatic malignancies in Ethiopian series (24,25,43). Conversely, thyroid and testicular cancers showed favorable survival, underscoring the potential for accessible, low-cost interventions to improve outcomes even in resource-limited settings (18). These observations emphasize the importance of preventive interventions, including HPV vaccination, hepatitis B and C control, and strengthened diagnostic and treatment capacity to reduce cancer mortality in LMICs (7).

The overall 5-year survival of 26.2% and median survival of 10 months observed in this cohort is markedly lower than pooled national estimates (~57%) and substantially below global benchmarks, reflecting the wide survival gap between Ethiopia and high-income countries(17,25). Previous Ethiopian studies on specific malignancies report rather poor long-term survival. For instance, a meta-analysis of breast cancer patients in Ethiopia found a pooled 5-year survival of ≈ 22% (95% CI: 8–40%), with median survival in individual cohorts ranging between ~ 10 to 58.7 months (44), while colorectal cancer cohorts in Addis Ababa documented median survival of 21 months and 5-year survival of 28.7% (45). These findings are broadly consistent with the poor survival reported here, though our larger and more heterogeneous cohort may better reflect national oncology outcomes. The prognostic importance of stage at diagnosis in our study, where early-stage disease conferred a median survival of 26 months versus only 8 months for advanced disease, aligns with both Ethiopian breast and colorectal cancer cohorts and global survival surveillance data from the CONCORD program, all of which demonstrate that late presentation remains the dominant driver of mortality(18,44,46). Treatment modality analysis further revealed a clear gradient of benefit, with median survival extending up to 21 months for patients receiving multimodal therapy, compared with 3 months for those without chemotherapy. This pattern mirrors international evidence that combined surgery, chemotherapy, and radiotherapy yield superior outcomes, but access in LMICs is constrained by limited infrastructure and financial barriers (7). Among systemic agents, paclitaxel and doxorubicin were associated with significant survival gains, while hormonal therapy produced the most favorable outcomes, consistent with prior Ethiopian breast cancer studies reporting a 57% reduction in mortality among patients receiving hormonal therapy (44). Finally, our multivariable analysis confirmed that advanced-stage disease doubled mortality risk (AHR 2.02, 95% CI 1.53–2.68), echoing both national and global findings that stage, treatment access, and social determinants of health are the strongest predictors of cancer survival (18,46,47).

### 3.1. Implications

#### Health system and policy implications

The findings carry urgent implications for Ethiopia’s cancer control strategy. First, expanding prevention and early detection must be prioritized. Scale-up of HPV vaccination and affordable screening for cervical cancer could avert a large proportion of cases (48,49). Similarly, introducing breast cancer awareness and early detection initiatives at the primary care level could shift stage distribution and improve survival. Second, equitable decentralization of cancer services is critical. Radiotherapy expansion, surgical oncology training, and regional oncology centers are essential to reduce delays and financial hardship. Third, ensuring reliable access to essential cancer medicines and diagnostics requires stronger procurement systems and integration of oncology into universal health coverage.

#### Research and global relevance

Beyond Ethiopia, these findings contribute to the evidence base on cancer in low-income countries, where robust survival data remain scarce. The cohort demonstrates that socioeconomic status and geography are powerful survival determinants, reinforcing the need for cancer control strategies that explicitly address equity. Our study also provides a baseline against which future interventions—such as HPV vaccination roll-out, radiotherapy scale-up, and health system reforms, can be evaluated.

### 3.2. Strengths and Limitations

This study leverages a large, well-characterized cohort, offering robust statistical power and the opportunity for subgroup analyses. Detailed clinical information, including stage at diagnosis, treatment modalities, and systemic therapy regimens, allowed identification of actionable survival determinants. Conducted at Ethiopia’s largest tertiary referral center, findings reflect national oncology patterns. Limitations include the single-center, retrospective design, incomplete records and follow-up bias, restricted molecular and biomarker data, and underreporting of recurrence or treatment-related complications. Absence of cost-effectiveness analyses limits guidance on prioritizing interventions in resource-constrained settings.

### 3.3. Conclusions

This study reveals strikingly poor survival outcomes among Ethiopian patients with solid tumors, largely driven by late presentation, limited treatment availability, and socioeconomic inequities. To our knowledge, it is the first comprehensive Ethiopian cohort to examine survival across major solid tumors and to demonstrate the independent prognostic effect of education and rural residence. Addressing these disparities through expanded prevention, early detection, decentralization of services, and equitable access to treatment is essential to improving outcomes. Strengthening cancer control in Ethiopia will not only benefit patients nationally but also inform strategies for comparable LMICs striving to close the global cancer survival gap.

## Figures and Tables

**Table 9:**
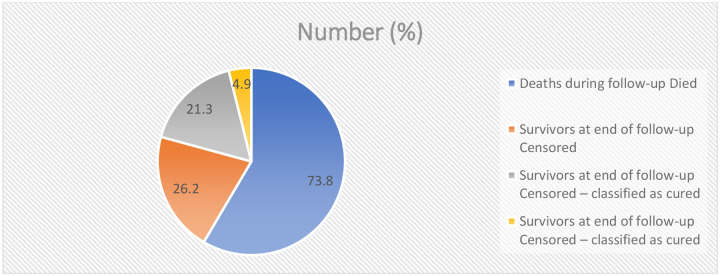
Overall, 5-year survival rates of patients with solid cancer at TASH (N = 1127)

**Table1:** Socio-demographic characteristics of study participants in TASH oncology center Addis Ababa (n = 1127)

Variable	Category	Number (%)
**Age (years)**	<30	134 (11.9)
	30–39	213 (18.9)
	40–49	257 (22.8)
	50–59	252 (22.4)
	60–69	194 (17.2)
	≥70	77 (6.8)
**Gender**	Female	743 (65.9)
	Male	384 (34.1)
**Region**	Addis Ababa	319 (28.3)
	Oromia	362 (32.1)
	Amhara	220 (19.5)
	SNNPR	145 (12.9)
	Other*	81 (7.2)
**Marital status**	Married	706 (62.6)
	Single	128 (11.4)
	Divorced	86 (7.6)
	Widowed	207 (18.4)
**Education**	Illiterate	534 (47.4)
	Primary	239 (21.2)
	Secondary	212 (18.8)
	Tertiary	142 (12.6)
**Occupational status**	Government	222 (19.7)
	Unemployed	477 (42.3)
	Private	428 (38.0)
**Occupational exposure**	Yes	304 (27.0)
	No	823 (73.0)
**Hospital cost**	Government	658 (58.4)
	Self	469 (41.6)
**Substance use**	Yes	180 (16.0)
	No	947 (84.0)

**Abbreviations: SNNPR** = Southern Nations, Nationalities, and Peoples’ Region.

Other* = Dire Dawa (11), Tigray (32), Somalia (10), Gumuz (5), Gambella (4), Harari (12), Afar (7)

**Table 2:** Clinical characteristics of solid tumor adult patients who had visited oncology center of TASH

Variables	Categories	Number (%)
Stage of cancer	Stage I	68(6.0)
	Stage II	150(13.3)
	Stage III	213(18.9)
	Stage IV	554(49.2)
	Unknown	142(12.6)
Comorbidity	Yes	219(19.4)
	No	908(80.6)
Type of comorbidity	Hypertension	47(4.2)
	Diabetic mellitus	22(2.0)
	HIV	65(5.8)
	Tuberculosis	28(3.0)
	Cardiac diseases	9(0.8)
	Diabetes and hypertension	17(1.5)
	Tuberculosis and HIV	3(0.3)
	Other*	28(2.5)
Medication use	Yes	338(30)
	No	789(70)
Type of medication	HAART	65(5.9)
	Antituberculosis	29(2.6)
	Antihypertensive	45(4.0)
	Anti-diabetic	21(1.9)
	Herbal medication	113(10.0)
	Antidiabetic and hypertensive	18(1.6)
	Antituberculosis and HAART	3(0.3)
	Other**	17(1.5)
Complication	Yes	276(24.5)
	No	851(75.9)
Type of complication	Pneumonia	27(2.4)
	Respiratory obstruction	26(2.3)
	Pleural effusion	46(4.1)
	Paralysis	38(3.4)
	Hypovolemic s hock	27(2.4)
	Sepsis	2(0.2)
	Renal complication	28(2.5)
	Thrombosis	30(2.7)
	Infection (Neutropenia)	27(2.4)
	Other***	25(2.8)
Recurrence	Yes	136(12.1)
	No	991(87.9)

**Abbreviations:** HIV: Human Immunodeficiency Virus, HARRT: Highly Active Antiretroviral Therapy. Comorbidities (Other*): Urinary tract infection (n=5), epilepsy (n=6), HepB+ve (n=4), HIV with DM (n=3), HIV with HTN (n=3), Medications (Other**): Anti-epileptic drugs (n=6), asthma treatment with nebulizers and steroids (n=5), combination therapy with HAART and anti-diabetic medications (n=3), HAART with anti-hypertensive medications (n=3) and Complications (Other***): Jaundice (n=7), ascites (n=5), osteoporosis (n=1), hepatotoxicity (n=1), raised ICP (n=3), PHTN (n=1), seizures (n=3).

**Table 3:** Median and overall survival of cancer patient at TASH (n=1127)

Variable	Category	Median survival time, months (95% CI)	5-year survival (%)	Log-rank χ^2^	p-value
Marital status	Overall	10.0 (8.96–11.04)	7.6	7.65	0.054
	Married	10.0 (8.74–11.26)	–		
	Single	8.0 (5.65–10.35)	–		
	Divorced	12.0 (5.53–18.47)	–		
	Widowed	11.0 (7.69–14.31)	–		
Environmental exposure	Overall	10.0 (8.96–11.04)	1.8	1.79	0.181
	No	11.0 (9.60–12.34)	–		
	Yes	8.0 (6.43–9.57)	–		
Stage of cancer	Stage I–II	26.0 (4.25–17.68)	10.0	31.95	<0.001**
	Stage III–IV	8.0 (6.88–15.20)	–		
Recurrence	No	9.0 (7.85–10.15)	10.0	14.59	<0.001**
	Yes	18.0 (14.96–21.04)	–		
Complication	No	10.0 (8.80–11.17)	10.0	2.34	0.013*
	Yes	9.0 (6.90–11.04)	–		
Treatment modality	Surgery	7.0 (5.15–8.85)	10.0	157.49	<0.001**
	Radiation	10.0 (6.92–13.08)	–		
	Surgery + Radiation	17.0 (3.81–30.19)	–		
	Surgery + Chemotherapy	16.0 (13.31–19.68)	–		
	Surgery + Chemotherapy + Radiation	21.0 (18.50–23.50)	–		
	Radiation + Chemotherapy	18.0 (15.28–20.72)	–		
	Chemotherapy only	9.0 (7.40–10.60)	–		
	Without chemotherapy	3.0 (2.48–3.53)	–		
Paclitaxel	Non-users	8.0 (6.88–9.14)	10.0	31.59	<0.001**
	Users	20.0 (16.27–23.74)	–		
Doxorubicin	Non-users	9.0 (7.87–10.13)	10.0	17.23	<0.001**
	Users	16.0 (11.03–20.97)	–		
Hormonal therapy	Non-users	9.0 (7.92–10.07)	10.0	38.16	<0.001**
	Users	24.0 (15.87–36.13)	–		

**Notes: CI**: Confidence Interval. *p* < 0.05 (*) indicates statistical significance; *p < 0.001 (**) indicates high statistical significance. Median survival times reflect the time at which 50% of patients were alive. Overall, 5-year survival represents the average survival across all categories for comparison.

**Table 4:** Cox regression with univariable and multi-variable result of cancer patients in TASH 2023. (n=1127)

Variable	Category	Censored (n)	Died (n)	Crude HR (95% CI)	Adjusted HR (95% CI)	p-value
Gender	Female	215	528	1.00	–	–
	Male	80	304	1.135 (0.985–1.307)	0.942 (0.792–1.121)	0.502
Marital status	Married	192	514	1.00	–	–
	Single	35	93	1.190 (0.953–1.485)	1.410 (1.105–1.799)	0.006*
	Divorced	30	56	0.843 (0.639–1.112)	0.903 (0.680–1.200)	0.483
	Widow	38	169	0.874 (0.734–1.041)	0.948 (0.787–1.142)	0.573
Exposure	No	220	573	1.00	–	–
	Yes	75	259	1.101 (0.951–1.276)	2.353 (1.568–3.530)	0.000**
Hospital cost	Government	177	481	1.00	–	–
	Self	118	351	1.166 (1.016–1.339)	1.049 (0.906–1.216)	0.521
Substance use	No	257	687	1.00	–	–
	Yes	38	145	1.201 (1.003–1.437)	1.146 (0.932–1.408)	0.196
Stage of cancer	Stage I & II	158	60	1.00	–	–
	Stage II&IV	88	679	1.983 (1.516–2.592)	2.020 (1.525–2.676)	0.000**
	Unknown	49	93	1.543 (1.113–2.141)	1.306 (0.923–1.847)	0.132
Recurrence	No	271	720	1.00	–	–
	Yes	24	112	0.688 (0.564–0.841)	0.791 (0.636–0.983)	0.034*
Complications	No	259	592	1.00	–	–
	Yes	36	240	1.120 (0.964–1.301)	1.233 (1.047–1.451)	0.012*
Treatment modality	Surgery	59	78	1.00	–	–
	Radiation	39	105	0.858 (0.639–1.151)	0.948 (0.697–1.290)	0.736
	Surgery + Radiation	21	39	0.713 (0.485–1.048)	0.739 (0.498–1.095)	0.132
	Surgery + Chemotherapy	96	128	0.576 (0.434–0.764)	0.674 (0.476–0.954)	0.026*
	Surgery + Radiation + Chemotherapy	45	60	0.567 (0.404–0.794)	0.713 (0.475–1.072)	0.104
	Radiation + Chemotherapy	20	82	0.630 (0.462–0.859)	0.677 (0.460–0.996)	0.047*
	Chemotherapy	11	130	0.962 (0.726–1.275)	1.050 (0.741–1.488)	0.785
	No chemotherapy	4	210	1.758 (1.353–2.284)	1.817 (1.382–2.389)	0.000**
Paclitaxel	No	231	710	1.00	–	–
	Yes	64	122	0.588 (0.484–0.714)	0.743 (0.586–0.942)	0.014*
Doxorubicin	No	212	715	1.00	–	–
	Yes	83	117	0.688 (0.566–0.838)	1.569 (1.033–2.384)	0.035*
Cyclophosphamide	No	220	741	1.00	–	–
	Yes	75	91	0.606 (0.487–0.754)	0.689 (0.420–1.131)	0.141
Fluorouracil	No	267	717	1.00	–	–
	Yes	28	115	0.801 (0.658–0.976)	0.745 (0.493–1.127)	0.164
Oxaliplatin	No	264	724	1.00	–	–
	Yes	31	108	0.865 (0.706–1.059)	1.420 (0.930–2.168)	0.104
Hormonal therapy	No	227	776	1.00	–	–
	Yes	68	56	0.444 (0.338–0.585)	0.592 (0.402–0.874)	0.008*
Capecitabine	No	282	807	1.00	–	–
	Yes	13	25	0.694 (0.465–1.034)	0.802 (0.519–1.239)	0.320

Notes: HR = hazard ratio. CI = confidence interval.

* p<0.05;

** p<0.001.

Reference category = HR 1.00.

## Data Availability

No datasets were generated or analyzed during the current study.

## List of Abbreviations

5-FU: 5-Fluorouracil
AHR: Adjusted Hazard Ratio
AIDS: Acquired Immunodeficiency Syndrome
CAPOX: Capecitabine and Oxaliplatin
CI: Confidence Interval
DM: Diabetes Mellitus
FAC: 5-Fluorouracil, Doxorubicin, and Cyclophosphamide
FOLFIRI: 5-Fluorouracil, Leucovorin, and Irinotecan
FOLFOX: 5-Fluorouracil, Leucovorin, and Oxaliplatin
HAART: Highly Active Antiretroviral Therapy
HBV: Hepatitis B Virus
HCV: Hepatitis C Virus
HIV: Human Immunodeficiency Virus
ICP: Intracranial Pressure
IE: Ifosfamide and Etoposide
LMICs: Low- and Middle-Income Countries
NPC: Nasopharyngeal Carcinoma
PHTN: Pulmonary Hypertension
RMS: Rhabdomyosarcoma
SDG: Sustainable Development Goal
SNNPR: Southern Nations, Nationalities, and Peoples’ Region
SPSS: Statistical Package for the Social Sciences
TASH: Tikur Anbessa Specialized Hospital
TNM: Tumor–Node–Metastasis
UTI: Urinary Tract Infection
WHO: World Health Organization
